# Human papillomavirus vaccination in men: a crucial step in Ecuadorian public health

**DOI:** 10.3389/fpubh.2026.1799490

**Published:** 2026-05-04

**Authors:** Harold A. Alexander-León

**Affiliations:** 1 Department of Research, SOLCA Núcleo de Quito, Quito, Ecuador; 2 Facultad de Ciencias de la Salud y Bienestar Humano, Departamento de Posgrados en Salud, Universidad Indoamérica, Quito, Ecuador; 3 Doctorado en Ciencias en Investigación Epidemiológica, Facultad de Administración y Salud Pública, Universidad Peruana Cayetano Heredia, Lima, Perú

**Keywords:** human papillomavirus viruses, men, papillomavirus infections, papillomavirus vaccines, sexual health

## Introduction

The most common sexually transmitted infection is human papillomavirus (HPV) infection (1). This oncogenic virus can infect the superficial skin of the anogenital and upper aerodigestive tracts (1). Sometimes the infection can persist, progress, and cause cancer, and is therefore attributed to approximately 5% of human cancers (1). Among the malignant lesions it produces are cervical and anogenital cancers, as well as oral and respiratory squamous cell carcinomas (1).

Most cases of cervical cancer worldwide are caused by persistent infections with one of the cancer-causing genotypes of HPV (2). According to the U.S. Centers for Disease Control and Prevention (CDC), the prevalence of genital HPV in adults aged 18 to 59 is approximately 40% in women and 45% in men (3). To prevent HPV infection from the most common high- and low-risk subtypes, the CDC recommends that both girls and boys receive HPV immunization (3). Vaccination between the ages of 9 and 12 can prevent approximately 90% of cervical cancers and precancerous lesions (2).

## Discussion

In Ecuador, cervical cancer has an incidence rate of 16 cases per 100,000 women and a mortality rate of 8.2 cases per 100,000 women, placing it in an intermediate position compared to other countries (4). However, the situation regarding cervical cancer in Quito, Ecuador's capital city, between 2015 and 2019, presents a concerning picture (4). The incidence rate in Quito has been higher than the global average (16.1 vs. 13.1 per 100,000 women), and the standardized mortality rate is also higher than the global average (10.4 vs. 7.1 per 100,000 women) (4). While the incidence trend has decreased over the years, the mortality rate has not shown significant change (4).

The observed stagnation in cervical cancer mortality rates in Quito, despite declining incidence, likely reflects multifactorial challenges within the healthcare system. Evidence from Ecuador indicates that delays in treatment initiation significantly impact survival: patients initially managed in the public health system experienced a mean delay of 162 days to start treatment compared to 62 days for those receiving comprehensive care at specialized centers, with advanced-stage patients (FIGO III–IV) showing the greatest survival disadvantage (HR: 0.48; 95% CI: 0.37–0.63) (5).

Additional contributing factors include fragmented healthcare delivery, limited screening coverage in rural and marginalized populations, and socioeconomic barriers that affect timely access to diagnosis and treatment (6, 7). While Ecuador has achieved high first-dose HPV vaccination coverage among girls, the protective effect of vaccination on cervical cancer mortality will require decades to manifest at the population level, given the long latency between HPV infection and invasive cancer. Therefore, strengthening secondary prevention, through equitable access to HPV testing, timely colposcopy, and prompt treatment of precancerous lesions, remains critical to reducing mortality in the short to medium term.

But a significant advance in Ecuador's public health has been achieved with the recent inclusion of boys in the HPV vaccination campaign, which took place between May and June 2024 and aimed to vaccinate approximately 595,000 children (8). This event represents an important step for the country and highlights Ecuador's role in the fight against HPV-related diseases. This step is crucial not only for men's health but also for the prevention of HPV-related infections in the general population.

To ensure the success of this initiative in Ecuador, it is essential to implement effective vaccine distribution and administration strategies continuously, supported by ongoing campaigns. This includes training healthcare workers, optimizing accessible vaccination centers, and implementing campaigns that promote public awareness and education. A crucial factor is vaccine acceptance to achieve adequate immunization. Furthermore, constant monitoring and evaluation of the vaccination program's implementation are essential. This will allow for continuous adjustments and improvements to vaccination strategies, guaranteeing their effectiveness and sustainability in the future.

Several ongoing improvements and forward-looking strategies are being implemented to strengthen Ecuador's HPV vaccination program and ensure its long-term sustainability. First, Ecuador is progressively transitioning toward a school-based delivery model (9), which has demonstrated superior coverage outcomes in regional peers: Peru and Mexico have achieved over 90% HPV vaccination coverage by integrating vaccination into the educational calendar and mandating reporting mechanisms (10).

Second, in alignment with the WHO's April 2024 position paper endorsing single-dose HPV vaccination schedules for individuals aged 9–20 years, Ecuador adopted in 2025 this simplified regimen on its immunization schedules for boys and girls aged nine years (11). This policy shift could reclassify children and adolescents who have received one dose as fully vaccinated, while reducing logistical burdens on the health system.

HPV vaccination coverage in men is exceptionally low (12). In 2019, only 4% of the male population was vaccinated against HPV (12). The vaccine's effectiveness has been high among men who had never been vaccinated against HPV (13). This supports the recommendation to vaccinate boys before they become sexually active in order to establish optimal protection through vaccination (13).

It is important to contextualize the burden of HPV-associated malignancies in men. In Ecuador, the estimated age-standardized incidence rates for HPV-related cancers in males include penile cancer (1.4 per 100,000 men), anal cancer (0.42 per 100,000 men), and oropharyngeal cancer (0.60 per 100,000 men) (14). Although these rates are lower than those observed for cervical cancer in women, they represent a preventable disease burden that disproportionately affects vulnerable populations. Globally, penile cancer incidence in Latin America ranges between 0.3 and 3.4 per 100,000 men, with higher rates associated with low socioeconomic status and limited access to preventive care (14, 15).

Regarding vaccine impact, a systematic review demonstrated that HPV vaccination in males confers high efficacy against precancerous lesions and anogenital disease (12). Vaccine efficacy against anal intraepithelial neoplasia grade AIN1 and AIN2/3 ranges from 91.1 to 93.1% and 89.6 to 91.7%, respectively. And efficacy against genital condyloma reaches 89.9% in HPV-naïve males (12). While long-term data on invasive cancer reduction in men are still emerging, mathematical models and real-world evidence from gender-neutral vaccination programs suggest that including boys in national immunization strategies accelerates herd immunity and reduces the overall circulation of oncogenic HPV types, thereby protecting both men and women (16).

**Figure 1:**
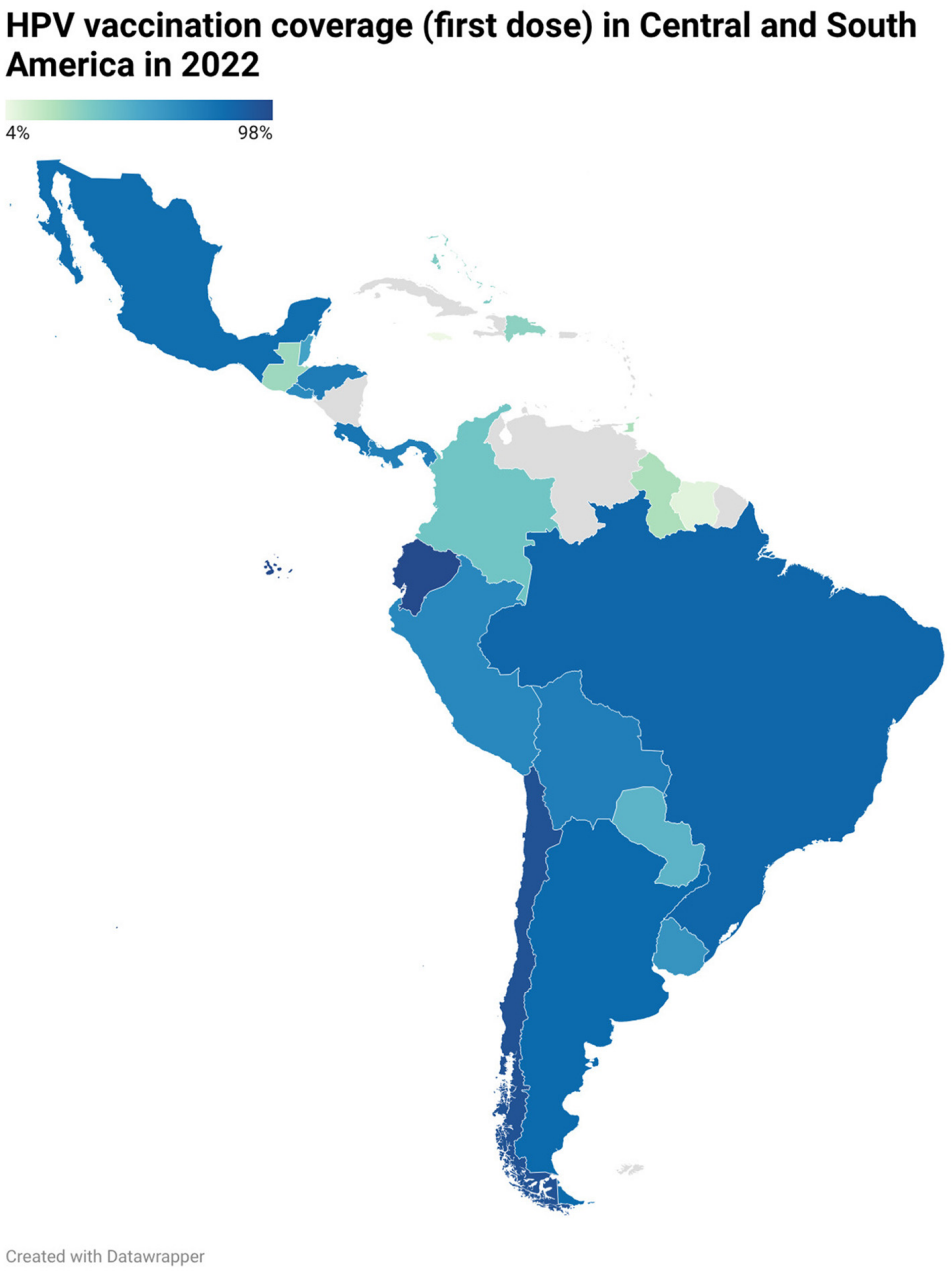
Human papillomavirus (HPV) vaccination coverage in Central and South America in 2022. Elaborated by the author based on information from PAHO, 2023 (17). Figure created with Datawrapper (25).

Fortunately, according to information available from the Pan American Health Organization (PAHO), Ecuador has the highest coverage of first-dose HPV vaccination in South America (17), with 98% coverage by 2022 (17). This places Ecuador first in the region for first-dose HPV vaccine coverage (17) (Figure 1).

Regarding the administration of the second dose, Ecuador faces a critical “completion gap” that undermines the effectiveness of its HPV vaccination program. Countrywide trend analysis from 2014 to 2024 reveals substantial volatility in HPV vaccination coverage: first-dose (HPV1) coverage, administered at age nine, declined from 98% in 2018 to just 28% in 2021, before rebounding to 98% in 2022 and stabilizing at 82% in 2023. However, second-dose (HPV2) coverage, administered six months later, has consistently lagged behind: it fell from 85% in 2018 to 24% in 2021, and although it improved to 52% by 2023, the persistent 30-percentage-point gap between HPV1 and HPV2 highlights ongoing challenges in ensuring series completion among adolescents. This disparity is further amplified at the provincial level, where logistical barriers, workforce shortages, and socioeconomic inequities disproportionately affect remote and marginalized communities (18).

Mathematical modeling studies are still needed to evaluate the effects of adding the male population to HPV vaccination programs (13). However, the impact on diseases attributed to the main HPV types (included in the vaccine) will be positive and fundamental for controlling these pathologies. But it will take several years to see the results of these expected reductions, given the age group covered by the vaccination programs.

It is important to mention that the Ministry of Public Health coordinates with the Ministry of Education to administer the HPV vaccine (8). However, a considerable proportion of the Ecuadorian population does not attend school (19). While the net enrollment rate in basic general education (population aged 5–14) has decreased by 4.7% in recent years (from 91.4 in 2007 to 96.1% in 2017), approximately 4% still do not attend basic general education (19). Therefore, this population group may be disadvantaged and require special attention.

To effectively reach Ecuadorian children and adolescents who do not attend school, a population disproportionately represented among rural, indigenous, and low-income households, targeted alternative delivery strategies are essential. Community-based vaccination campaigns, led by trained community health workers, can significantly improve coverage among out-of-school populations (20). This can be determined using data obtained from monitoring the vaccination program and compared in the future with the incidence of HPV-related diseases in different population groups.

Additionally, in Ecuador, regardless of the person's sex, in January 2026 the Ministry of Public Health incorporated people diagnosed with acquired immunodeficiency virus between 15 and 45 years of age as a target population of the HPV vaccination strategy (21). The inclusion of people living with HIV (PLWH) as a priority group for HPV vaccination in Ecuador addresses a critical epidemiological need. As of 2023, approximately 51,000 individuals were living with HIV in Ecuador, with an annual incidence of 1,900 cases for 2023, and significant disparities by sex and geographic region (22). PLWH face increased risk of developing HPV-associated malignancies, including anal, cervical, vulvar, and oropharyngeal cancers (23).

Regarding vaccine impact, WHO guidelines recommend a three-dose (where possible) HPV vaccination schedule for immunocompromised individuals, including PLWH, based on evidence of acceptable immunogenicity and safety in this population (24). Although seroconversion rates in PLWH may be modestly lower than in immunocompetent individuals, HPV vaccination remains highly immunogenic and is not associated with increased adverse events. Looking forward, integrating HPV vaccination into routine HIV care in Ecuador, through coordinated delivery at specialized HIV treatment centers and community-based testing sites, could substantially reduce the future burden of HPV-related cancers among PLWH.

In conclusion, the inclusion of HPV vaccination for men in national immunization campaigns is a significant step forward in the fight against HPV-related diseases in Ecuador. This step will not only benefit men but also contribute to overall public health by reducing the burden of HPV-related diseases in the Ecuadorian population.
